# Genetic testing for hereditary breast cancer in Poland: 1998–2022

**DOI:** 10.1186/s13053-023-00252-6

**Published:** 2023-06-13

**Authors:** Jacek Gronwald, Cezary Cybulski, Tomasz Huzarski, Anna Jakubowska, Tadeusz Debniak, Marcin Lener, Steven A Narod, Jan Lubinski

**Affiliations:** 1 International Hereditary Cancer Center, Department of Genetics and Pathology, Pomeranian Medical University, Szczecin, Poland; 2 Dalla Lana School of Public Health, University of Toronto, Toronto, ON Canada; 3 Womens College Research Institute, Toronto, ON Canada

**Keywords:** BRCA1, BRCA2, Breast Cancer, Genetic testing

## Abstract

BRCA1 and BRCA2 mutations contribute to both breast cancer and ovarian cancer worldwide. In Poland approximately 4% of patients with breast cancers and 10% of patients with ovarian cancer carry a mutation in *BRCA1*. The majority of mutations consist of three founder mutations. A rapid inexpensive test for these three mutations can be used to screen all Polish adults at a reasonable cost. In the region of Pomerania of North-western Poland nearly half a million tests have been performed, in large part through engaging family doctors and providing ready access to testing through the Pomeranian Medical University. The following commentary provides a history of genetic testing for cancer in Pomerania and the current approach to facilitating access to genetic testing at the Cancer Family Clinic for all adults living in the region.

Poland is a country of 38,000,000 inhabitants who occupy 121,000 square miles of Central Europe. Poland is a genetically homogeneous country inhabited primarily by ethnic Slavs. Each year approximately 25,000 Polish women are diagnosed with breast cancer and 5,000 are diagnosed with ovarian cancer (Globocan). Approximately 4% of patients with breast cancer and 10% of patients with ovarian cancer carry a mutation in *BRCA1* [1, 2]. The number of patients undergoing genetic testing is currently unknown.

Genetic testing for breast cancer susceptibility was introduced in Poland in 1995, shortly after the discoveries of the *BRCA1* and *BRCA2* genes in 1994 and 1995, respectively. Clinical and research activity originated in Northwest Poland at the Cancer Family Clinic at the Pomeranian Medical University in Szczecin, which remains a very active centre today.

The Cancer Family Clinic predated the cloning of *BRCA1* and *BRCA2*. Cancer families have been registered here since 1992. In the early days, at the first clinic visit, pedigree information and demographic details were collected and risks were based on family histories. The genetics laboratory was established in 1995 and clinical testing ensued. In 1998, the University spearheaded a national campaign, supported by grants from the National Ministry of Health and the European Community to identify families at high risk of carrying mutations in hereditary cancer genes, including those responsible for breast-ovarian cancer, hereditary colon cancer and several rare syndromes. Local cancer registries identified women with breast or ovarian cancer and (men and women) with colon cancer and invited them to come to a designated testing centre where a pedigree was taken and genetic testing was offered. In recent years, the invitations have come from the treating physicians and not the registries. In 2002, genetic testing was offered free of charge to readers of the Polish women’s magazine Twoj Styl through a coupon feature [3].

The volume of testing was greatly enhanced by the identification of three founder alleles in *BRCA1* (5382insC, C61G, 4153delA) which account for approximately 80% of the pathogenic *BRCA1* mutations found throughout Poland [4]. The majority of patients were tested only for founder mutations - full gene sequencing was reserved for those with strong family histories. The clinical activity expanded through the establishment of a network of affiliated hospital-based clinical cancer centers located throughout the country – clinics were accessible to more than half of the total population. The clinics were staffed by local administrators and a key group of four clinician-scientists –based in Szczecin - who were responsible for counselling patients and obtaining samples for testing in the affiliated clinics (Table 1). The process of testing and counselling at these clinics followed the same procedure as that of the central Family Cancer Clinic in Szczecin. To date, approximately half of the mutations identified have been through clinics outside of Szczecin.

**Table 1:** Network of cancer family clinics closely collaborating with Hereditary Cancer Centre in Szczecin in 2022

NO	CITY	DOCTORS
1.	Białystok	Posmyk R., Leśniewicz R., Lubiński J.
2.	Bielsko-Biała	Wiśniowski R., Lubiński J.
3.	Brzozów	Jaśkiewicz J., Gronwald J.
4.	Kielce	Siołek M., Góźdź S., Lubiński J.
5.	Konin	Stawicka M., Huzarski T.
6.	Koszalin	Gronwald J., Cybulski C.
7.	Łomża	Dach K., Huzarski T.
8.	Olsztyn	Huzarski T.
9.	Opole	Tomiczek-Szwiec J., Huzarski T.
10.	Poznań	Godlewski D., Stawicka M., Mądry R., Cybulski C.
11.	Rzeszów	Kluz T., Gronwald J.
12.	Siedlce	Prusaczyk A., Gronwald J.
13.	Szczecin - leading	Gronwald J. Huzarski T. Cybulski J. Dębniak T. Ashuryk O., Lubiński J.
14.	Świdnica/Wałbrzych	Kilar E., Pyka P., Huzarski T.
15.	Toruń	Jarkiewicz-Tretyn J., Cybulski C., Huzarski T.
16.	Warszawa/ Otwock/ Wieliszew	Huzarski T.
17.	Zielona Góra	Szwiec M., Sibilski R. Huzarski T.

Typically, the attending physicians attended a one- or two-day clinic each week, where between 50 and 200 patients are seen and tested. In 2000–2001 family doctors from West-Pomeranian region collected approximately one million cancer family histories from 75% of the inhabitants of the region and women with at least one relative affected by breast or ovarian cancer were offered testing for the *BRCA1* founder mutations. From 1998 to 2022, blood was taken from approximately 500,000 individuals and tested in the central laboratory in Szczecin. 9,012 BRCA1 mutation carriers have been identified to date.

In 1998, a research registry for female BRCA1 carriers was established in collaboration with Dr Steven Narod at Women’s College Hospital in Toronto which continues to accrue female patients with pathogenic mutations identified in Poland. At present, nine countries participate in the registry and over 5,000 BRCA1 carriers from Poland have been enrolled [5]. In 1996, Dr Lubinski established a complementary biobank for these patients which obtains and stores blood specimens from women in the research registry, including whole blood, serum and plasma.


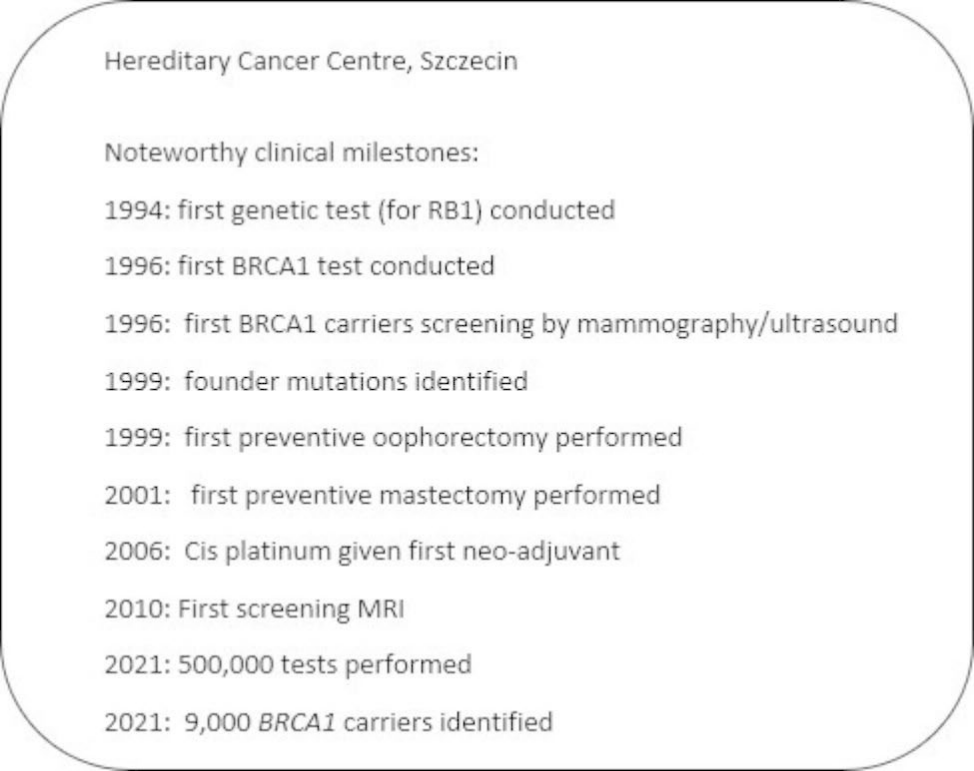


The genetic testing process is transparent and streamlined in order to minimise barriers.

The goal is to identify as many mutation carriers as possible Women with a new diagnosis of breast or ovarian cancer are identified through their treating physician and invited to the local cancer genetics testing center. At the appointment a family history is taken and the process and consequences of testing are explained. Consent for research is obtained. A blood sample for is taken for testing. The genetic test result (for founder mutations) is returned in four days and before treatment is begun. The appointment takes approximately 20 min.

If a mutation is found, relatives are invited for testing. The proband is given a paper sheet with details of how the relative can pursue testing. If a woman does not have cancer or a family history of cancer, but expresses a wish to be tested, she is accommodated. At present, approximately one-quarter of the patients who are tested have a personal history of cancer and three quarters are unaffected. Testing is free. If a mutation is found the doctor sees the patient in a follow-up clinic twice a year. Women with a *BRCA1* mutation are recommended to have a bilateral salpingo-oophorectomy at age 35 in accordance with international guidelines [6]. Those who do not have an oophorectomy are offered trans-vaginal ultrasound screening twice a year. A breast screening MRI is offered once a year. The risks and benefits of tamoxifen and preventive bilateral mastectomy are discussed. All of the screening tests and surgical interventions are at no cost to the patient. The average appointment takes ten to 15 min.

Of the 9012 individuals identified in the database with a *BRCA1* mutation, 7,388 are women (82%). Of these, 5,143 (70%) are enrolled in the registry-based prospective study (representing 30% of all the individuals in the international registry). Of these, 2,525 women had cancer at baseline and 2,618 women had no cancer at baseline. Of the 2,618 women who were initially cancer-free, 859 have since developed cancer (423 breast cancers; 303 ovarian cancers, 170 other cancers). There are currently 1,759 cancer-free women under observation in the database.

By several measures (in particular, the number of carriers identified) the Polish cancer genetics program has been a success. It is of interest to speculate why this might be. First, the primary goal is to identify as many female carriers of *BRCA1* mutations as possible. This is facilitated by the presence of the founder effects in Poland, which make testing inexpensive and the results easy to interpret. Mutations in *BRCA2* and other susceptibility genes are comparatively rare [7]. *CHEK2* mutations are common to be sure, but the clinical utility of finding these mutations is not well-established [8, 9]. There is little enthusiasm about finding mutations in the various genes which populate the common test panels in North America. Unlike the situation in North America, the choice of genes to be included is decided by the caregivers and not by the commercial laboratories. Physicians are not hampered by the need to explain to patients the clinical and reproductive implications of being a heterozygous carrier of mutations in genes such as e.g. *MUTYH*. Testing has been centralised since the outset and most tests are conducted in a single laboratory. The system is hierarchical - there are no collaborators in the strict sense - counselling and testing is done by our in-house team of clinician-scientists who travel by train to satellite clinics and a small number of affiliated clinicians (Table 1). The clinics do not employ genetic counsellors - who typically spend more time with clients than do physicians - thereby limiting the number of counselees. Women who are found to have a mutation are offered follow-up visits with the same physician at no charge. MRI screenings and preventive surgeries are covered by national health insurance. The patients have expressed a high degree of satisfaction in the testing and counselling process.

## Data Availability

The data presented in this study are available from the corresponding author on reasonable request.
